# Tickborne Relapsing Fever in Israel

**DOI:** 10.3201/eid1111.050521

**Published:** 2005-11

**Authors:** Gil Sidi, Nadav Davidovitch, Ran D. Balicer, Emilia Anis, Itamar Grotto, Eli Schwartz

**Affiliations:** * Jacobi Medical Center, Bronx, New York, USA; † Medical Corps, Israel Defense Forces, Israel; ‡ Ministry of Health, Jerusalem, Israel; § Sheba Medical Center and Tel Aviv University, Tel Aviv, Israel

**Keywords:** relapsing fever, tickborne infections, caves, Israel, Ornithodoros, epidemiology, dispatch

## Abstract

We evaluated the epidemiology of relapsing fever from 1971 to 2003 in Israel. In civilians, incidence declined from 0.35 to 0.11 cases per 100,000 persons annually; in military personnel it averaged 6.4 cases per 100,000 persons annually. These data imply that the pathogen and vector continue to exist in Israel.

Relapsing fever is caused by infection with *Borrelia* species that can genetically vary their surface antigens, which causes repeated stimulation of the immune system and recurring episodes of fever. Two distinct types of relapsing fever exist, louseborne relapsing fever and tickborne relapsing fever (TBRF) (*1**,**2*). The TBRF *Borrelia* sp. is transmitted to humans by an infected *Ornithodoros* tick bite (*2*).

Human TBRF is generally contracted only in the geographic range of the tick vectors. *Ornithodoros* species of ticks belong to the *Argasidae* (soft tick) family that feeds nocturnally. The primary reservoirs of *Borrelia* spp. are rodents and lagomorphs (*2*). Many *Ornithodoros* species have been reported worldwide, each with its characteristic *Borrelia* species (*2**–**5*).

After an incubation period of ≈7 days, TBRF typically begins abruptly with fever, chills, headache, myalgia, arthralgia, and abdominal pain. Death is rare. Diagnosis is usually made by observing spirochetes on peripheral blood smears. Antimicrobial drug treatment is occasionally associated with the Jarisch-Herxheimer reaction (*6*).

Since 1946, all cases of relapsing fever described in Israel have been tickborne. *B. persica*, transmitted by *Ornithodoros tholozani* is thought to be the cause of TBRF in Israel. The *O. tholozani* tick was first identified in 1937 and is prevalent in Middle Eastern and central Asian countries. It is primarily found in dark, moist areas such as caves and abandoned buildings (*7*). In Israel, TBRF has traditionally been called cave fever because of the known correlation to cave exposure (*8*). The only survey conducted in Israel described the incidence of TBRF from 1954 to 1967 and concluded that the incidence of TBRF was declining (*9*).

## The Study

In Israel, all cases of TBRF in civilians are reported to the Ministry of Health and investigated by an epidemiologist nurse. Cases in soldiers are reported to the military health branch of the Surgeon General Headquarters of the Israel Defense Force. An epidemiologic investigation of cases in soldiers is conducted by the reporting physician. We evaluated the demographic, clinical, and geographic data from reports of civilians from 1971 to 2003 and of soldiers from 1975 to 2003.

We included confirmed cases, in which a person had both febrile illness and a positive peripheral blood smear, and associated cases, in which a person had a relapsing febrile illness and was associated with a confirmed case. Tests for trends were conducted with PEPI software, version 4.0 (Sagebrush Press, Salt Lake City, UT, USA). A total of 606 cases were reported during the study years, 283 in civilians, and 323 in soldiers.

**Figure 1:**
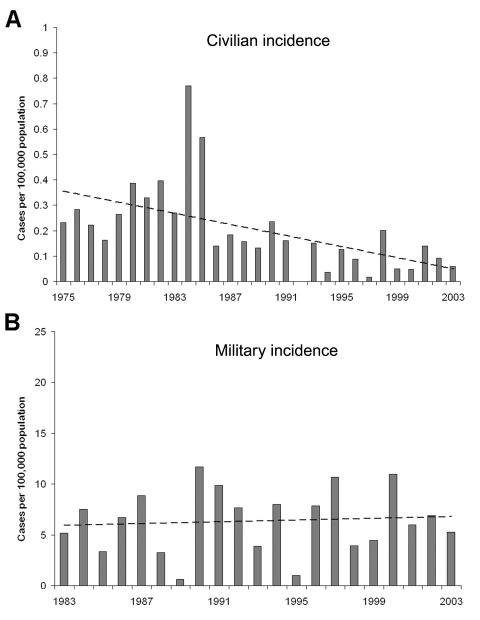
Incidence of tickborne relapsing fever in Israel. A) among civilians, 1975–2003; B) among soldiers, 1983–2002. Dotted line indicates prevalence.

The yearly civilian incidence is shown in Figure 1A. The yearly incidence average in civilians declined from 0.35 cases per 100,000 population from 1975 to 1985 to 0.11 cases per 100,000 population from 1986 to 2003 (p<0.001). The peak in civilian cases in 1984 and 1985 was unexplained; 2 cases occurred in immigrants from Ethiopia 3 months after they arrived in Israel. The incidence of TBRF in the military from 1983 to 2003 has been relatively constant, with an average of 6.4 cases per 100,000 population annually (Figure 1B).

The patients included 529 men (87%) and 77 women (13%). Of 359 patients for whom this information was available, 304 (85%) were of Jewish origin and 55 (15%) were of non-Jewish origin, similar to the proportions in the Israeli population. Information on exposure circumstances was available for 256 cases. The most commonly reported exposures were visiting caves (64%), visiting abandoned buildings (5%), and engaging in other outdoor activity (19%). In 12% of the cases, exposure to caves was excluded, but the exposure circumstances were not identified. In soldiers, 43% of cases were associated with outdoor exposure, usually related to prolonged periods of lying on the ground. In civilians, 49% were students and 16% were outdoor workers. One hundred cases occurred in clusters with an average of 3.3 (range 2–10) people in each cluster.

## Conclusions

Soldiers comprised more than half of the total cases in this study, with an average incidence 36 times greater than in civilians. The higher incidence in soldiers may be due to increased risk of exposure because of their specific activities or better case reporting in the Israel Defense Force.

During the last century in industrialized countries, a significant decline in many infectious diseases has been observed (*10*). This decline can be attributed to elimination or significant reduction of pathogens or decreased exposure to the pathogens.

**Figure 2:**
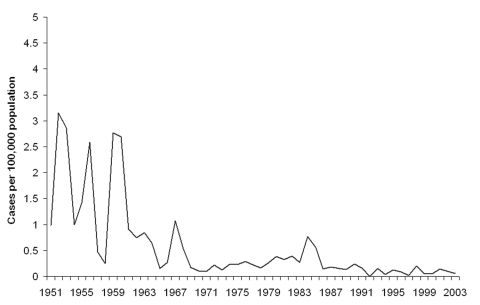
Civilian yearly incidence of tickborne relapsing fever, Israel, 1951–2003.

This study has shown a decrease in the incidence of TBRF in civilians, with no similar decrease in soldiers. This decrease becomes even more evident when compared to the incidence in Israel during the 1950s and 1960s (Figure 2). That the incidence has remained similar over time among military personnel indicates that the pathogen and the vector continue to exist in Israel and that soldiers are at a high risk of contracting it because of their activities. Infection has occurred although Israel Defense Force commanders are aware of TBRF and all soldiers and commanders receive annual guidelines on preventing TBRF. Furthermore, entering caves is prohibited, and most TBRF cases in the military (67%) were not due to cave exposure. The declining incidence among civilians is likely due to a decrease in the rate of exposure with increased urbanization. A civilian subpopulation that continues to be at risk is schoolchildren; most cases occurred after field trips.

Although TBRF was reported throughout the year, most cases occurred during summer and fall. Possible explanations include higher *Ornithodoros* tick activity, higher human outdoor activity, and lighter clothes worn during these seasons. TBRF can be contracted throughout Israel, despite the varying climatic conditions (Table). In soldiers, 63% of cases were contracted in the hot, arid, southern regions of Israel. The high number of cases in the southern regions is probably because most military training activity is conducted there. In civilians, more cases were contracted in the northern and central regions of Israel where more persons travel. That 64% of the cases are associated with caves might be explained by the relatively stable conditions in caves that are favorable to ticks.

**Table:** Geographic distribution (%) of tickborne relapsing fever cases

Geographic region	Civilians	Military	Total
Israel			
North	36	18	29
Center	50	12	35
South	10	63	32
Other			
Africa	4	0	2
Lebanon	0	7	3
Total			100

Most cases that occurred in the military were in men. Although women also served in the Israeli military, only a fraction of them served in positions that required outdoor exposure. The illness was also more preponderant in civilian men. This tendency has been observed in previous reports, and likely results from men’s high-risk behavior.

In conclusion, TBRF continues to be endemic in Israel and still poses a considerable hazard for soldiers and civilians. Although TBRF has been substantially reduced in civilians, the incidence in soldiers has not declined. Sufficient information on the habits of the *O. tholozani* tick and its reservoirs is not available. To reduce the incidence of TBRF, further studies are warranted to accurately map *Ornithodoros* tick infestations. Further studies are also needed to identify *Borrelia* reservoirs in Israel and to study the habits of its vector.
